# Real-World Outcomes of Nivolumab Plus Ipilimumab in Metastatic Melanoma: A Stratified Analysis of First- and Second-Line Treatment

**DOI:** 10.3390/cancers18121994

**Published:** 2026-06-18

**Authors:** Alexandr Iurchenkov, Anastasia Danilova, Polina Shilo, Vladimir Stoliarov, Polina Rakhmanova, Ilia Kanner, Anna Basharina, Daniil Stroyakovskiy

**Affiliations:** 1 Moscow City Oncology Hospital No. 62, 143423 Moscow, Russia; dr.yurchenkov@gmail.com (A.I.); anastasia.danilova@gmail.com (A.D.); polzarakhmanova@yandex.ru (P.R.); ilyakanner@gmail.com (I.K.);; 2 Lahta Clinic, 197229 Saint Petersburg, Russia; polinashilo0@gmail.com; 3 Ledin Clinic, 121609 Moscow, Russia; vladimir.stoliarov1@gmail.com; 4 Pfizer Innovation LLC, 123112 Moscow, Russia

**Keywords:** metastatic melanoma, nivolumab, ipilimumab, real-world evidence, immune checkpoint inhibitors

## Abstract

**Background**: Immune checkpoint inhibitors have significantly improved survival in metastatic melanoma. Combination nivolumab plus ipilimumab demonstrated superior efficacy in randomized trials, including CheckMate 067, but data beyond the first-line setting remain limited. This study evaluated real-world outcomes and predictors of response across different lines of therapy, with an exploratory comparison between first- and second-line use. **Methods**: This retrospective single-center study included patients with metastatic melanoma treated with nivolumab plus ipilimumab as first- or second-line therapy at Moscow City Oncology Hospital No. 62 between September 2015 and October 2023. Eligible patients had histologically confirmed melanoma and received at least one cycle of dual immune checkpoint blockade. Clinical and demographic data were extracted from electronic medical records. The primary endpoints were progression-free survival (PFS) and overall survival (OS); secondary endpoints included objective response rate (ORR) and safety. Survival outcomes were estimated using the Kaplan–Meier method and compared using the log-rank test. Multivariable Cox proportional hazards models adjusted for clinically relevant covariates were applied to evaluate the association between treatment line and survival outcomes. Additional prognostic analyses were performed using backward stepwise multivariable Cox regression. **Results**: Median follow-up was 18.2 months (IQR, 6.7–30.4). Median PFS in the overall cohort was 7.9 months (95% CI, 4.2–11.5), and median OS was not reached (NR); 5-year OS: 50%. The ORR was 45.8%, including 15.1% complete responses. Median PFS was 9.0 months (95% CI, 5.0–12.9) in first-line and 6.1 months (95% CI, 3.4–8.8) in second-line patients. Median OS was NR in the first-line cohort and was 30.5 months (95% CI: NR) in the second-line cohort. In exploratory analyses, OS did not differ significantly between patients treated in the first-line (*n* = 141) versus second-line setting (*n* = 63) (*p* = 0.848). After adjustment for clinical and demographic characteristics, line of therapy was not associated with OS (HR 0.93; 95% CI, 0.58–1.50; *p* = 0.762). Immune-related adverse events were associated with longer PFS (HR 0.66; 95% CI, 0.46–0.93), although this may reflect time-dependent bias. **Conclusions**: Nivolumab plus ipilimumab demonstrated clinically meaningful activity in both first- and second-line settings. Outcomes were numerically lower than in clinical trials, consistent with broader real-world populations. In exploratory analyses, OS did not differ significantly between treatment lines after adjustment for clinical and demographic characteristics. These findings should be interpreted with caution given the retrospective design and potential sources of bias.

## Simple Summary

Metastatic melanoma is a serious and often life-threatening form of skin cancer, but recent advances in immunotherapy have improved patient outcomes. One commonly used treatment combines two drugs, nivolumab and ipilimumab, which help the immune system attack cancer cells. However, most evidence about this combination comes from clinical trials that include selected patients and may not reflect everyday clinical practice. In this study, we aimed to evaluate how effective and safe this treatment is in real-world patients, including those with more complex disease. We also sought to identify factors that may influence treatment success. Our findings provide practical insights into how this therapy performs outside of clinical trials and may help doctors make better treatment decisions and improve outcomes for patients with metastatic melanoma.

## 1. Introduction

The introduction of immune checkpoint inhibitors has markedly improved outcomes in metastatic melanoma [1,2,3,4]. The final 10-year analysis of the CheckMate 067 trial reports a median overall survival (OS) of 71.9 months for patients treated with combination immunotherapy using nivolumab (anti-PD-1) and ipilimumab (anti-CTLA-4), compared to 36.9 months with nivolumab monotherapy and 19.9 months with ipilimumab alone [5]. These results confirm the superior long-term efficacy of the combination regimen. However, this benefit comes at the cost of increased toxicity: grade 3–4 treatment-related adverse events occurred in 63% of patients receiving the combination, versus 25% and 30% with nivolumab and ipilimumab monotherapy, respectively [5]. Based on these data, nivolumab plus ipilimumab has become a standard-of-care first-line treatment for advanced melanoma, as reflected in current clinical practice guidelines from the National Comprehensive Cancer Network and the European Society for Medical Oncology [6].

It is important to recognize that these findings are derived from a randomized controlled trial with a carefully selected patient population, which may not reflect the full spectrum of clinical complexity encountered in routine practice [7]. Real-world cohorts often include patients with poor prognostic factors, such as brain metastases, which were present in only 3.5% of patients in the combination arm of CheckMate 067 [2]. In contrast, such patients are frequently encountered in clinical practice and merit focused investigation.

Moreover, although combination immune checkpoint blockade is approved for first-line use, its efficacy in later lines of therapy remains incompletely characterized in prospective trials. The SWOG S1616 trial represents the only randomized prospective study addressing this question to date in patients with metastatic melanoma progressing on prior anti-PD-1 therapy. In this phase II study, nivolumab plus ipilimumab significantly improved progression-free survival (PFS) compared with ipilimumab monotherapy (median PFS 3.0 vs. 2.5 months; HR 0.63; *p* = 0.04). The combination was also associated with a higher objective response rate (ORR) (28% vs. 9%) and more durable responses, with approximately 34% of patients remaining progression-free at 6 months compared with 13% in the ipilimumab group [8].

Complementing these data, real-world evidence further supports the activity of nivolumab plus ipilimumab after progression on prior anti-PD-(L)1 therapy. In the multicentre retrospective cohort study by Pires da Silva et al., 193 of 355 patients with metastatic melanoma resistant to anti-PD-(L)1 received ipilimumab plus anti-PD-1. In this subgroup, treatment was associated with an ORR of 31%, median PFS of 3.0 months, and median OS of 20.4 months, with grade 3–5 adverse events observed in 31% of patients. These findings indicate that a subset of patients may derive clinically meaningful benefit from dual checkpoint blockade even after failure of prior PD-1-based therapy [9].

The optimal sequencing of systemic therapy is particularly relevant in patients with BRAF-mutant melanoma. Based on the results of the DREAMseq and SECOMBIT trials, upfront combination immunotherapy is generally considered the preferred first-line approach in patients without rapidly progressive or symptomatic disease. Importantly, immune checkpoint blockade retains clinically relevant activity when administered after prior BRAF-targeted therapy, supporting the need to evaluate outcomes across treatment lines [10,11,12].

Thus, according to the NCCN guidelines (v2.2026), nivolumab plus ipilimumab is included among the preferred regimens for second-line treatment of metastatic melanoma, alongside nivolumab plus relatlimab, anti-PD-1 monotherapy, and BRAF/MEK inhibitors in patients with BRAF-mutant disease [6].

In routine clinical practice, organizational and system-level constraints may limit access to combination immunotherapy in the first-line setting. Consequently, the real-worldeffectiveness of this approach when administered in the second line represents a clinically relevant question.

In this study, we assess the clinical outcomes of patients with metastatic melanoma treated with standard-dose combination immunotherapy (anti-CTLA-4 plus anti-PD-1) in a real-world setting stratified by line of therapy. We further aim to identify clinical and biological predictors of response to inform future patient stratification and therapeutic decision-making. An exploratory comparison between treatment lines was performed and should be interpreted with caution, given the potential for immortal time bias inherent to analyses of later-line therapies.

## 2. Methods

This retrospective, single-center study included patients with metastatic melanoma treated with combination immunotherapy comprising nivolumab and ipilimumab as first- or second-line therapy at Moscow City Oncology Hospital No. 62 between September 2015 and October 2023.

Eligible patients had histologically confirmed melanoma and received at least one cycle of dual immune checkpoint blockade with anti-CTLA-4 and anti-PD-1 antibodies. No formal exclusion criteria were applied beyond missing key clinical data required for outcome assessment. Clinical data were extracted from electronic medical records.

The study was carried out in accordance with the Declaration of Helsinki. The study protocol was approved by the Ethics Committee of Moscow City Oncology Hospital No. 62. All participants gave their informed consent for data collection and analysis prior to the initiation of treatment.

Collected demographic and clinical variables included age, sex, melanoma subtype, BRAF mutation status, disease presentation (de novo metastatic vs. initially localized), serum lactate dehydrogenase (LDH) levels, presence of brain metastases, and overall metastatic burden. High metastatic burden was defined as the presence of ≥4 metastatic sites. Treatment-related variables comprised line of therapy, number of administered immunotherapy cycles, and occurrence of immune-related adverse events (irAEs).

Tumor response was assessed using radiological imaging and evaluated according to the immune-Related Response Evaluation Criteria In Solid Tumors (iRECIST) [13]. Patients were followed by an medical oncologist at each cycle of immunotherapy. Radiological assessments included computed tomography performed in all patients every 2–3 months. Brain magnetic resonance imaging was performed in all patients at treatment initiation; thereafter, in patients with known brain metastases, MRI was repeated every 2–3 months, while in patients without baseline brain involvement, MRI was performed upon clinical suspicion of intracranial disease progression.

The primary endpoints were PFS and OS. PFS was defined as the time from initiation of nivolumab plus ipilimumab to documented disease progression or death from any cause, whichever occurred first. OS was defined as the time from treatment initiation to death from any cause. For comparisons between treatment lines, OS was calculated from initiation of first-line therapy. Patients without events were censored at the date of last follow-up. Secondary endpoints included ORR, defined according to iRECIST, and safety, assessed based on the occurrence of irAEs. irAEs were evaluated through clinical assessment at each visit, as well as biochemical, hormonal, and instrumental investigations, in accordance with institutional protocols for immunotherapy safety monitoring. Assessment of irAEs was performed according to the Common Terminology Criteria for Adverse Events 5.0 (CTCAE) in the context of routine clinical practice [14].

Survival outcomes were estimated using the Kaplan–Meier method and compared using the log-rank (Mantel–Cox) test. Categorical variables were analyzed using Pearson’s χ^2^ test or Fisher’s exact test, as appropriate. To assess the association between line of therapy (first vs. second line) and OS, a Cox proportional hazards model adjusted for clinically relevant covariates was applied. The model included age, sex, BRAF mutation status, prior adjuvant anti-PD-1 therapy, serum LDH levels, high metastatic burden (≥4 metastatic sites), and presence of brain metastases. Separately, to explore prognostic factors associated with survival outcomes, univariate Cox regression analyses were performed, followed by multivariable modeling using a backward stepwise selection procedure based on the Wald statistic. Variables with *p* < 0.10 in univariate analysis were considered for inclusion in the multivariable model. Hazard ratios (HRs) with 95% confidence intervals (CIs) were reported.

Given the retrospective design, the analysis was subject to potential selection bias and confounding by indication. Comparisons between treatment lines were considered exploratory due to potential immortal time bias.

All statistical analyses were performed using IBM SPSS Statistics, version 26.0 (IBM Corp., Armonk, NY, USA). All tests were two-sided, and *p*-values < 0.05 were considered statistically significant.

## 3. Results

### 3.1. Patient Characteristics

Between September 2015 and October 2023, 205 patients with metastatic melanoma received combination immunotherapy with nivolumab plus ipilimumab at Moscow City Oncology Hospital No. 62. The median follow-up was 18.2 months (range, 0.1–110; interquartile range (IQR), 6.7–30.4). Baseline clinical and demographic characteristics are summarized in Table 1.

Overall, the study population was representative of patients with advanced melanoma treated in routine clinical practice. Patients were broadly distributed across clinically relevant subgroups, including BRAF mutation status and disease presentation (de novo metastatic versus initially localized disease). Combination nivolumab plus ipilimumab was administered as first-line therapy in 141 patients and as second-line therapy in 63 patients. One additional patient received treatment in the third-line setting and was included only in the analysis of the overall cohort.

Baseline characteristics were generally well balanced between treatment lines. However, brain metastases were more frequently observed in patients receiving second-line therapy (44.4% vs. 29.0%, *p* = 0.05), while no other clinically meaningful differences were identified.

Among those treated in the second-line setting, prior first-line regimens for metastatic disease most commonly included anti-PD-1 monotherapy (*n* = 29, 46.0%), combined BRAF and MEK inhibition (*n* = 26, 40.7%), triplet therapy with BRAF, MEK, and PD-L1 inhibitors (*n* = 4, 6.3%), and other regimens, including BRAF inhibitor monotherapy or chemotherapy (*n* = 4, 6.3%). Adjuvant anti-PD-1 therapy for localized melanoma prior to the development of metastatic disease had been administered in 16 patients (7.8%).

The median number of administered combination cycles was 4 (IQR, 2–4), with 58.1% of patients completing all four planned induction cycles. Maintenance nivolumab monotherapy was continued in 23.9% of patients, with a median of 15 maintenance cycles (IQR, 9–24.5). At the time of data cutoff, 39.0% of patients had died.

### 3.2. Efficacy of Combination Nivolumab and Ipilimumab in the Overall Cohort

**Figure 1:**
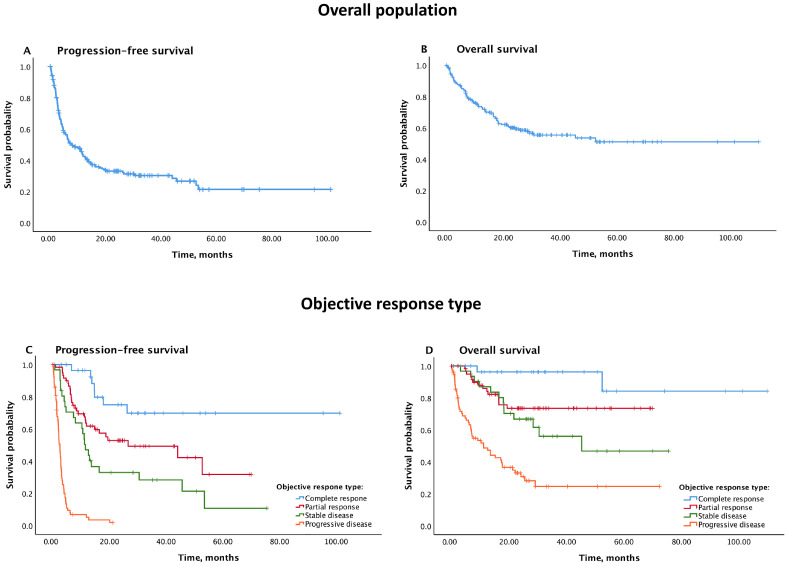
Kaplan–Meier survival analyses in overall population. Progression-free survival (**A**) and overall survival (**B**) in the overall cohort. Survival stratified by objective response: PFS (**C**) and OS (**D**). CR, complete response; PR, partial response; SD, stable disease; PD, progressive disease.

In the overall population, the median PFS was 7.9 months (95% CI, 4.2–11.5; Figure 1A). The median OS was not reached (NR) (Figure 1B), with cumulative OS rates of 74% at 1 year, 60% at 2 years, and 50% at 5 years.

The ORR was 45.8%, including complete response (CR) in 15.1% and partial response (PR) in 30.7% of patients. Stable disease (SD) and progressive disease (PD) were observed in 15.6% and 38.5% of patients, respectively (Table 2). The associations between clinicodemographic characteristics and ORR are presented in Table S1.

Median PFS differed significantly across response categories, being NR for CR, 26.6 months for PR (95% CI, 0.1–54.8), 11.5 months for SD (95% CI, 8.9–14.1), and 2.5 months for PD (95% CI, 2.0–3.0) (Figure 1C). A similar pattern was observed for OS, with median OS not reached for CR and PR, 45.3 months for SD (95% CI, NR), and 11.2 months for PD (95% CI, 4.9–17.4) (Figure 1D).

Among 69 patients with brain metastases, 48 received radiotherapy. Median OS was 36.9 months (95% CI, 0.4–47.9) in the radiotherapy group, compared with 24.2 months (95% CI, 11.5–79.2) in patients who did not receive radiotherapy (*p* = 0.092).

### 3.3. Safety

IrAEs were documented in 120 patients (58.5%) during treatment with combination nivolumab and ipilimumab. Grade 3–4 toxicity was observed in 23 patients (8.9%). Treatment discontinuation due to immune-related toxicity occurred in 37 patients (18%). As this analysis reflects real-world clinical practice, and given the limitations of routine medical documentation, some irAEs may not have been recorded, and in certain cases, severity grading was unavailable. Consequently, the incidence of grade 3–4 toxicity is likely underestimated in the present study.

### 3.4. Prognostic Factors for Survival Outcomes

In univariate Cox regression analysis, the occurrence of irAEs was associated with improved PFS (HR 0.69; 95% CI, 0.49–0.97), whereas baseline brain metastases (HR 1.64; 95% CI, 1.15–2.33) and prior exposure to anti-PD-1 therapy (HR 1.45; 95% CI, 1.01–2.10) were associated with worse PFS.

In multivariate analysis, irAEs (HR 0.66; 95% CI, 0.46–0.93) and de novo metastatic disease at presentation (HR 0.65; 95% CI, 0.43–0.98) remained independently associated with improved PFS. Negative prognostic factors included baseline brain metastases (HR 1.76; 95% CI, 1.23–2.53), prior anti-PD-1 therapy (HR 1.52; 95% CI, 1.02–2.26; *p* = 0.039), and BRAF mutation status, which showed a borderline association (HR 1.36; 95% CI, 0.99–1.88) (Table 3).

For OS, baseline brain metastases were the only factor significantly associated with worse outcomes in both univariate and multivariate analyses (HR 1.62; 95% CI, 1.03–2.54) (Table 4). Female sex (HR 1.49; 95% CI, 0.95–2.35) and the occurrence of irAEs (HR 0.66; 95% CI, 0.42–1.04) showed trends toward association with OS but did not reach statistical significance.

### 3.5. Outcomes in the First-Line Treatment Cohort

Among the 141 patients who received nivolumab plus ipilimumab as first-line therapy, the median follow-up was 18.4 months (range, 0.1–95.0; IQR, 7.3–29.6). Baseline clinical and demographic characteristics are summarized in Table 1.

**Figure 2:**
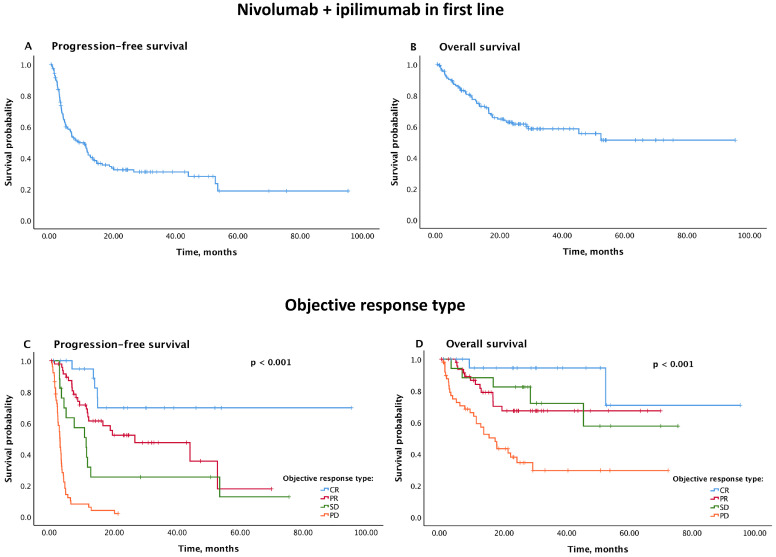
Kaplan–Meier survival analyses for the first-line nivolumab + ipilimumab cohort. Progression-free survival (**A**) and overall survival (**B**) in the first-line treatment cohort. Survival stratified by objective response: PFS (**C**) and OS (**D**). CR, complete response; PR, partial response; SD, stable disease; PD, progressive disease.

The median PFS was 9.0 months (95% CI, 5.0–12.9) (Figure 2A). Cumulative PFS rates at 1, 2, and 5 years were 43%, 33%, and 20%, respectively. Median OS was NR (Figure 2B), with corresponding OS rates of 78% at 1 year, 62% at 2 years, and 50% at 5 years.

The ORR was 51.1%, including CR in 15.6% and PR in 35.5% of patients. Stable and progressive disease were observed in 12.1% and 36.9% of patients, respectively (Table 2).

Median PFS was NR among patients achieving CR. For patients with PR, SD, and PD, median PFS was 26.6 months (95% CI, 5.0–48.2), 11.1 months (95% CI, 3.9–18.3), and 2.8 months (95% CI, 2.2–3.4), respectively (Figure 2C). Median OS was NR for patients with CR, PR, or SD, whereas patients with PD had a median OS of 19.3 months (95% CI, 11.8–26.9) (Figure 2D).

IrAEs occurred in 87 patients (61.7%). Grade 3–4 irAEs were observed in 17 patients (12.1%).

Discontinuation of first-line combination therapy occurred due to disease progression in 34.0% of patients, treatment-related toxicity in 21.3%, and patient refusal or organizational reasons in 11.3%; 33.3% remained on treatment at data cutoff.

Among patients treated in the first-line setting, 73 (52.1%) completed all four induction cycles, while 67 (47.8%) received fewer than four cycles. Among patients who discontinued treatment early, the most common reason was disease progression (23, 34.3%), followed by immune-related toxicity (19, 28.4%); in 7 patients (10.4%), discontinuation was due to organizational reasons, while in 19 (28.4%), the reason was not documented.

Patients who completed all four induction cycles demonstrated a trend toward improved outcomes compared with those who received fewer than four cycles. Median PFS was 11.9 months (95% CI, 8.1–15.7) versus 4.7 months (95% CI, 1.8–7.8), respectively (*p* = 0.070). Median OS was NR in patients who completed four cycles, compared with 45.3 months (95% CI, 8.5–82.2) in those who received fewer than four cycles. These findings should be interpreted with caution due to the potential for immortal time bias.

Results of univariate and multivariable Cox regression analyses identifying prognostic factors for PFS and OS in the first-line cohort are presented in the Supplementary Materials (Supplementary Tables S2 and S3).

### 3.6. Outcomes in the Second-Line Treatment Cohort

A total of 63 patients received nivolumab plus ipilimumab as second-line therapy (Table 1). The median follow-up duration was 19.4 months (range, 0.1–70.1; IQR, 6.7–32.7).

**Figure 3:**
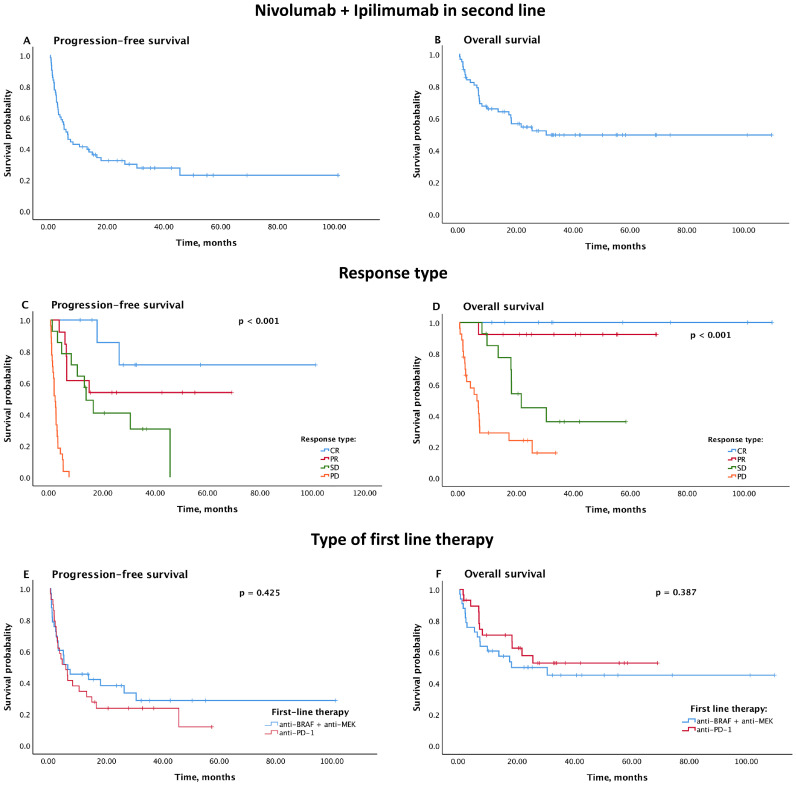
Kaplan–Meier survival analyses for the second-line nivolumab + ipilimumab cohort. Progression-free survival (**A**) and overall survival (**B**) in the second-line treatment cohort. Survival stratified by objective response: PFS (**C**) and OS (**D**). Survival stratified by previous first-line therapy: PFS (**E**) and OS (**F**).

The median PFS was 6.1 months (95% CI, 3.4–8.8) (Figure 3A), with cumulative PFS rates of 41% at 1 year and 23% at 4 years. Median OS was 30.5 months (95% CI, NR) (Figure 3B), with 1-, 2-, and 4-year OS rates of 65%, 54%, and 49%, respectively.

An objective response was achieved in 34.9% of patients, including CR in 14.3% and PR in 20.6%. SD and PD were observed in 22.2% and 42.9% of patients, respectively (Table 2).

Median PFS according to response category was NR for patients with CR or PR, whereas patients with SD and PD had median PFS values of 13.6 months (95% CI, 8.0–19.2) and 1.8 months (95% CI, 0.8–2.8), respectively (Figure 3C). Median OS was NR for patients with CR or PR; among those with SD and PD, median OS was 21.7 months (95% CI, 8.5–35.0) and 6.1 months (95% CI, 2.7–9.5), respectively (Figure 3D).

IrAEs occurred in 33 (53.1%) of patients. Grade 3–4 toxicity was observed in 6 patients (9.4%).

In the second-line setting, treatment discontinuation was most frequently due to progression (62.5%), followed by toxicity (10.9%) and refusal or logistical factors (7.9%), while 18.8% of patients were still receiving therapy.

Among patients treated in the second-line setting, 38 (59.4%) completed all four induction cycles, while 26 (40.6%) received fewer than four cycles. Notably, among patients who discontinued treatment early, the vast majority (24/26, 92.3%) stopped therapy due to disease progression, whereas only one patient (3.8%) discontinued due to immune-related toxicity.

Patients who completed all four induction cycles demonstrated markedly improved outcomes compared with those who received fewer than four cycles. Median PFS was 26.2 months (95% CI, 7.3–45.4) versus 1.4 months (95% CI, 1.0–1.7), respectively (*p* < 0.001). Median OS was NR in patients who completed four cycles, compared with 6.1 months (95% CI, 2.9–9.4) in those who received fewer than four cycles. When stratified by type of prior systemic therapy, no significant differences in PFS or OS were observed between subgroups (Figure 3E,F).

Results of univariate and multivariable Cox regression analyses identifying prognostic factors for PFS and OS in the second-line cohort are presented in the Supplementary Materials (Supplementary Tables S4 and S5).

### 3.7. Comparative Efficacy of Nivolumab + Ipilimumab Dual Immunotherapy in First- Versus Second-Line

**Figure 4:**
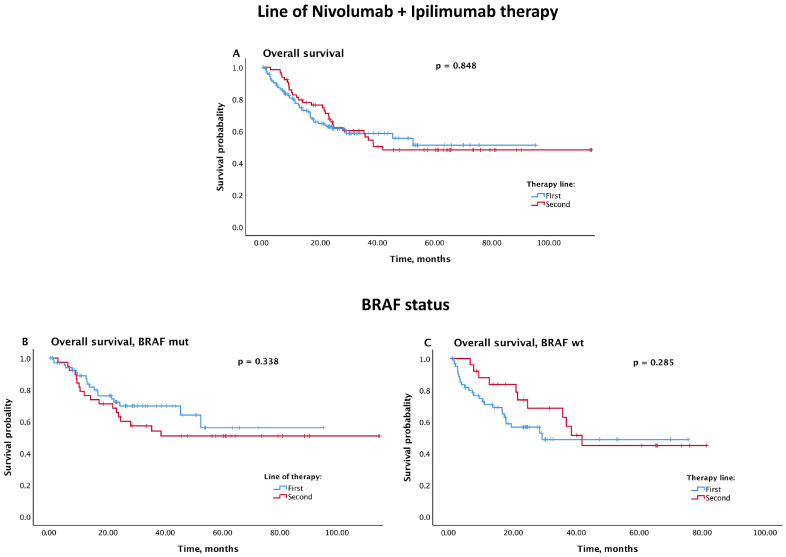
Comparative efficacy of nivolumab + ipilimumab dual immunotherapy in first- versus second-line settings OS (**A**) according to the line of combination therapy (first-line vs. second-line nivolumab + ipilimumab). OS by BRAF mutation status: BRAF-mutant (**B**) and BRAF wild-type (**C**). Mut, mutated; wt, wild type.

OS did not differ significantly between patients receiving nivolumab plus ipilimumab as first-line versus second-line therapy. Median OS (calculated from initiation of first-line therapy) was NR in the first-line cohort and was 41.9 months (95% CI, NR) in the second-line cohort (*p* = 0.848; Figure 4A). In multivariable analysis with adjustment for clinically relevant covariates, line of therapy was not associated with OS (HR 0.93; 95% CI, 0.58–1.50; *p* = 0.762).

In patients with BRAF-mutant melanoma, median OS was NR in either the first-line (*n* = 71) or second-line (*n* = 38) cohorts, with no significant difference observed between treatment settings (*p* = 0.338; Figure 4B).

Among patients with BRAF wild-type melanoma, median OS was 29.1 months (95% CI, NR) in the first-line cohort (*n* = 63) and 41.8 months (95% CI, 32.4–51.3) in the second-line cohort (*n* = 25); this difference did not reach statistical significance (*p* = 0.285; Figure 4C).

## 4. Discussion

In this retrospective real-world cohort of patients with metastatic melanoma treated with nivolumab plus ipilimumab, we observed clinically meaningful outcomes in the combined cohort, with results numerically lower than those reported in pivotal trials, potentially reflecting differences in patient population, such as the inclusion of second-line patients and a higher prevalence of brain metastases. In the first line setting median PFS was 7.9 months and median OS was NR, with a 5-year OS rate of 50% (compared with a median PFS of 11.5 months and a 5-year OS rate of approximately 52% in CheckMate 067). In exploratory analyses stratified by line of therapy, OS did not differ significantly between first- and second-line groups after adjustment, and irAEs were associated with longer PFS (HR 0.66; 95% CI, 0.46–0.93); however, both findings should be interpreted with caution due to potential selection bias, immortal time bias, and time-dependent bias. Outcomes were numerically more favorable in the first-line setting. Baseline brain metastases consistently predicted worse outcomes. Overall, these findings highlight the effectiveness of dual immune checkpoint blockade in routine clinical practice, while underscoring the gap between real-world and trial-based outcomes.

This study represents the first large real-world cohort of Russian patients with metastatic melanoma treated with combined nivolumab and ipilimumab. Preliminary findings from this cohort were previously presented in abstract form at ASCO 2025 [15].

The clinical and demographic characteristics observed provide a valuable reflection of real-world patients with cutaneous melanoma in Russia. Consistent with nationwide molecular-epidemiological data [16], 53.2% of our cohort harbored BRAF mutations, aligning with reported frequencies in this population and exceeding those in international randomized trials (32% in CheckMate-067 [5]). Brain metastases were present in 33.7% of patients, substantially higher than in CheckMate-067 (4%) [5]. Notably, similar rates (~30%) have been reported in other real-world analyses of combined immune checkpoint blockade, supporting the representativeness of our cohort [17]. The cohort also demonstrated near-equal sex distribution (50.7% male, 49.3% female vs. 65% male in CheckMate-067). This balance reflects our real-world setting and contrasts with other RWE studies of dual immunotherapy, where male predominance was more common [18,19]. Such differences underscore the importance of evaluating outcomes in clinically heterogeneous populations outside the context of randomized trials.

Across the entire cohort, dual immune checkpoint blockade demonstrated effectiveness comparable to international RWE studies [18,19], with an ORR of 45.8% and median PFS of 7.9 months. First-line treatment outcomes were more favorable (ORR 51.1%, median PFS 9.0 months), closely aligning with CheckMate-067 and CheckMate-511 [5,19], reinforcing the role of nivolumab plus ipilimumab as a standard-of-care option in treatment-naïve patients, as reflected in current ESMO and NCCN guidelines.

With regard to second-line therapy, the efficacy observed in our cohort (ORR 34.9%, median PFS 6.1 months, median OS 30.5 months) is consistent with previously published real-world studies and aligns with the SWOG S1616 trial—the only prospective randomized study in this setting [8]. In patients with BRAF-mutant melanoma, these outcomes are also comparable to those reported in the DREAMseq trial arm in which patients received first-line BRAF-targeted therapy followed by immunotherapy [10,11]. Importantly, although no statistically significant differences in OS were observed between first- and second-line settings after adjustment, this finding should be interpreted cautiously given the retrospective design and potential biases. Taken together, these data suggest that dual immune checkpoint blockade may retain activity beyond the first-line setting in selected patients, but careful patient selection remains essential.

Depth of response remained a strong determinant of survival. Complete or partial responders experienced markedly prolonged PFS and OS, consistent with long-term analyses from CheckMate-067 and other immunotherapy studies [5,19]. While real-world assessment may be influenced by imaging schedules and clinical decision-making, the reproducibility of this association across studies supports its clinical validity.

The occurrence of irAEs was associated with improved PFS and a trend toward longer OS, corroborating prior reports in melanoma and other solid tumors [20,21,22,23]. Although retrospective data limited detailed grading and timing, these observations are biologically plausible, reflecting enhanced immune activation. At the same time, emerging evidence suggests that early or organ-specific irAEs may carry distinct prognostic implications [24], warranting further prospective evaluation.

Several factors were associated with less favorable prognosis. Elevated LDH, a marker of tumor burden and impaired antitumor immunity [25,26], correlated with inferior outcomes. BRAF mutation status was associated with shorter PFS, consistent with meta-analytic evidence of its negative prognostic value [27]. Nevertheless, prospective sequencing trials, including SECOMBIT and DREAMseq, demonstrated superior long-term outcomes when immunotherapy precedes targeted therapy in BRAF-mutant melanoma, supporting upfront combination immunotherapy in appropriately selected patients [11,12,28].

High tumor burden—defined as >4 metastatic lesions—was associated with poorer OS (median 24.16 months vs. NR in patients with fewer metastases). This aligns with prior studies linking tumor burden to aggressive disease biology and reduced immunotherapy efficacy. Mechanistically, larger tumors are associated with profound local and systemic immunological alterations, including enrichment of immunosuppressive cells and increased inhibitory immune mediators, which collectively promote an immunosuppressive tumor microenvironment. Consideration of tumor burden within this framework may inform patient stratification and the development of combination strategies to overcome immune resistance [29].

Brain metastases emerged as one of the strongest negative prognostic factors, particularly in the second-line setting. Although upfront nivolumab plus ipilimumab demonstrates durable intracranial activity [18], outcomes were substantially poorer following prior systemic therapy, reflecting more advanced disease. In our cohort, the use of radiotherapy in patients with brain metastases was associated with numerically improved survival. These findings highlight the importance of a multidisciplinary treatment approach, integrating systemic immunotherapy with local modalities such as radiotherapy in selected patients, and support the early use of combination immunotherapy in patients with brain metastases [29,30].

In our cohort, female sex showed a trend toward association with worse OS (HR 1.49; 95% CI, 0.95–2.35; *p* = 0.083), although this did not reach statistical significance. Interestingly, this observation contrasts with findings from a Danish DAMMED study, in which female sex was associated with improved outcomes, suggesting that the impact of sex on survival with dual immune checkpoint blockade remains inconsistent across datasets. The reasons for these divergent results are not yet clear and may reflect differences in patient populations, prior treatments, or underlying biological factors, underscoring the need for further investigation into sex-based differences in immunotherapy response [31,32].

Taken together, our findings appear consistent with the broader real-world literature on nivolumab plus ipilimumab in metastatic melanoma. Our results are numerically higher than those reported in the Polish multicentre cohort (ORR 34%, Disease Control Rate 58%, median PFS 6.3 months, with OS median NR at 12 months). They are broadly consistent with the large international cohort by Serra-Bellver et al., which demonstrated an overall ORR of 48%, median PFS of 7.9 months, and median OS of 38 months. In this study, more favorable outcomes were observed in selected subgroups—particularly treatment-naïve patients without brain metastases—who achieved an ORR of 56.6% and a median PFS of 13.7 months, highlighting the impact of baseline disease characteristics on treatment efficacy. Notably, outcomes in our second-line cohort appear numerically more favorable than those reported by Pires da Silva et al. in patients resistant to anti-PD-(L)1 therapy (ORR 31%, median PFS 3.0 months, median OS 20.4 months), although such cross-study comparisons should be interpreted with caution given differences in patient selection and potential biases. Overall, these comparisons suggest that our outcomes fall within the expected range of real-world efficacy, while reflecting the heterogeneity of patient populations and treatment settings [9,17,33].

This study has several important limitations inherent to its retrospective, observational design. The absence of randomization and a control group limits the ability to draw causal inferences, and the findings should therefore be interpreted as descriptive associations observed in routine clinical practice. In addition, treatment allocation was not controlled and may have been influenced by clinical factors such as disease burden, prior therapy, and physician judgment, introducing potential selection bias. The inclusion of both first- and second-line treatment settings further contributes to clinical heterogeneity, and although stratified analyses were performed, residual confounding cannot be excluded. Furthermore, performance status was not systematically captured during data collection and therefore could not be included in the analysis. Data on LDH were incomplete, reflecting real-world clinical practice where this parameter is not consistently assessed in all patients. Despite these limitations, the study provides clinically relevant real-world data reflecting treatment patterns and outcomes in an unselected patient population, including patients with adverse prognostic features who are often underrepresented in clinical trials.

## 5. Conclusions

In this real-world cohort of patients with metastatic melanoma, nivolumab plus ipilimumab demonstrated clinically meaningful activity, with outcomes numerically lower than those reported in clinical trials, likely reflecting differences in patient population, including a higher prevalence of brain metastases and the inclusion of pretreated patients. Outcomes were more favorable in the first-line setting, although comparisons across treatment lines should be interpreted with caution. Baseline disease characteristics, particularly brain metastases and tumor burden, were strongly associated with survival. While irAEs were associated with improved PFS, this finding should be interpreted cautiously given the retrospective design and incomplete characterization of toxicity. Overall, these data support the effectiveness of dual immune checkpoint blockade in routine clinical practice, while highlighting the importance of patient selection and optimal treatment sequencing.

## Figures and Tables

**Table 1:** Demographic and Disease Characteristics of the Patients at Baseline.

Characteristics	All Patients (*n* = 205)	First-Line (*n* = 141)	Second-Line (*n* = 63)	*p*-Value (1 L vs. 2 L)
Median age, years (range)	55 (19–84)	57 (19–82)	52 (26–84)	0.10 *
Age Groups, *n* (%)				0.18
<40	36 (17.6)	25 (17.7)	11 (17.5)	
40–65	119 (58.0)	75 (53.2)	43 (68.3)	
>65	50 (24.4)	41 (29.1)	9 (14.3)	
Sex, *n* (%)				0.39
Male	104 (50.7)	69 (49)	35 (55.6)	
Female	101 (49.3)	72 (51)	28 (44.4)	
Melanoma type, *n* (%)				0.21
Cutaneous	167 (81.5)	110 (78.0)	57 (89.1)	
Mucosal	7 (3.4)	6 (4.3)	1 (1.6)	
Uveal	6 (2.9)	6 (4.3)	0	
Unknown primary site	25 (12.2)	19 (13.5)	6 (9.4)	
Abundance of pigmentation, *n* (%)				0.49
Pigmented Melanoma	184 (89.8)	128 (90.8)	56 (87.5)	
Amelanotic melanoma	21 (10.2)	13 (9.2)	8 (12.5)	
BRAF-status, *n* (%)				0.52
BRAFwt	88 (42.9)	63 (44.7)	25 (39.7)	
BRAFmut	109 (53.2)	71 (50.4)	37 (58.7)	
Unknown ^‡^	8 (3.9)	7 (4.9)	1 (1.6)	
Disease stage at initial diagnosis, *n* (%)				0.57
Local	141 (68.8)	95 (67.4)	45 (71.4)	
Metastatic	64 (31.2)	46 (32.6)	18 (28.6)	
LDH, *n* (%)				0.31 ^†^
Patients tested	125 (61.0)	86 (61.0)	38 (60.3)	
of whom >ULN	54 (43.2)	39 (45.3)	14 (36.8)	
of whom ≤ULN	71 (56.8)	47 (54.7)	24 (63.2)	
CNS metastasis at baseline				0.05
Yes	69 (33.7)	41 (29.0)	28 (44.4)	
No	136 (66.3)	100 (71.0)	35 (55.6)	
Number of metastatic lesions				0.18
≥4	69 (33.7)	43 (30.5)	25 (40%)	
<4	136 (66.3)	98 (69.5)	38 (60%)	
Prior systemic therapy for metastatic disease, *n* (%)				
Anti-PD-1 monotherapy			29 (46.0)	
Anti-BRAF + Anti-MEK Inhibitors			26 (40.7)	
Anti-BRAF + Anti-MEK + Anti-PD-L1 Inhibitors			4 (6.3)	
Other			4 (6.3)	

LDH: Lactate dehydrogenase; I-O: Immuno-oncology; ULN: Upper limit of normal. Percentages within the subgroups were calculated based on the number of patients who received testing for that baseline characteristic. * Mann–Whitney U test; ^†^ *p*-value only for patients with tested LDH level; ^‡^ the corresponding status was not specified in the medical records.

**Table 2:** The best overall tumor response.

	All Patients (*n* = 205)	First Line (*n* = 141)	Second Line (*n* = 63)
ORR, *n* (%)	94 (45.8%)	72 (51.1%)	22 (34.9%)
Complete response	31 (15.1%)	22 (15.6%)	9 (14.3%)
Partial response	63 (30.7%)	50 (35.5%)	13 (20.6%)
Stable disease	32 (15.6%)	17 (12.1%)	14 (22.2%)
Progressive disease	79 (38.5%)	52 (36.9%)	27 (42.9%)

ORR—objective response rate.

**Table 3:** Univariate and multivariate Cox proportional hazards regression model analysis of PFS in patients with metastatic melanoma (overall cohort).

Variables		Median PFS, Mos (95% CI)	Univariable Analysis		Multivariable Analysis	
			HR (95% CI)	*p*-Value	HR (95% CI)	*p*-Value
Age	≤55 (*n* = 102)	7.9 (2.2–13.5)	1.14 (0.81–1.60)	0.456		
	>55 (*n* = 102)	8.4 (4.2–12.6)				
Sex	Male (*n* = 104)	11.1 (8.4–15.3)	1.14 (0.81–1.61)	0.437		
	Female (*n* = 100)	7.07 (2.0–12.1)				
BRAF status	BRAFwt (*n* = 88)	11.1 (6.2–16.0)	1.23 (0.9–1.66)	0.184	1.36 (0.99–1.88)	**0.055**
	BRAFmut (*n* = 108)	6.6 (1.1–12.0)				
LDH	≤ULN (*n* = 71)	11.5 (2.3–20.8)	1.07 (0.87–1.31)	0.455		
	>ULN (*n* = 53)	4.3 (2.8–5.8)				
I-O toxicity	No (*n* = 84)	5.4 (2.6–8.3)	0.69 (0.49–0.97)	**0.032**	0.66 (0.46–0.93)	**0.019**
	Yes (*n* = 120)	11.9 (4.9–18.8)				
Number of metastatic lesions	<4 (*n* = 136)	10.6 (5.9–15.2)	1.27 (0.89–1.81)	0.186		
	≥4 (*n* = 69)	4.9 (2.5–7.2)				
CNS metastasis at baseline	No (*n* = 136)	11.6 (6.3–16.9)	1.64 (1.15–2.33)	**0.006**	1.76 (1.23–2.53)	**0.002**
	Yes (*n* = 69)	4.9 (2.4–7.3)				
I-O adjuvant/metastatic therapy	No (*n* = 155)	11.4 (6.05–16.88)	1.45 (1.01–2.10)	0.047	1.52 (1.02–2.26)	**0.039**
	Yes (*n* = 49)	6.2 (3.81–8.64)				
Disease stage at initial diagnosis	Local (*n*= 140)	6.57 (2.19–10.95)	0.74 (0.50–1.08)	0.120	0.65 (0.43–0.98)	**0.034**
	Metastatic (*n*= 64)	11.17 (5.3–17.05)				

CNS: central nervous system; LDH: Lactate dehydrogenase; I-O: Immuno-oncology; ULN: Upper limit of normal. Statistically significant values (*p* ≤ 0.05) are highlighted in bold.

**Table 4:** Univariate and multivariate Cox proportional hazards regression model analysis of OS in patients with metastatic melanoma (overall cohort).

Variables		Median OS, Mos (95% CI)	Univariable Analysis		Multivariable Analysis	
			HR (95% CI)	*p*-Value	HR (95% CI)	*p*-Value
Age	≤55 (*n* = 102)	NR	1.35 (0.86–2.10)	0.187		
	>55 (*n* = 102)	28.4 (20.0–36.8)				
Sex	Male (*n* = 104)	NR	1.35 (0.86–2.11)	0.184	1.49 (0.95–2.35)	0.083
	Female (*n* = 100)	52.5 (95% CI: NR)				
BRAF status	BRAFwt (*n* = 88)	29.1 (95% CI: NR)	0.97 (0.65–1.45)	0.871		
	BRAFmut (*n* = 108)	NR				
LDH	≤ULN (*n* = 71)	NR	1.13 (0.87–1.46)	0.350		
	>ULN (*n* = 53)	52.4 (2.4–102.5)				
I-O toxicity	No (*n* = 84)	25.5 (0.1–52.7)	0.69 (0.44–1.08)	0.104	0.66 (0.42–1.04)	0.071
	Yes (*n* = 120)	NR				
Number of metastatic sites	<4 (*n* = 136)	NR	1.50 (0.96–2.35)	0.074		
	≥4 (*n* = 69)	25.5 (5.2–45.8)				
CNS metastasis	No (*n* = 136)	NR	1.58 (1.01–2.48)	**0.044**	1.62 (1.03–2.54)	**0.035**
	Yes (*n* = 69)	25.5 (11.8–39.2)				
I-O adjuvant/metastatic therapy	No (*n* = 135)	NR	0.97 (0.58–1.60)	0.895		
	Yes (*n* = 69)	NR				
Disease stage at initial diagnosis	Local (*n*= 140)	NR	0.75 (0.45–1.25)	0.273		
	Metastatic (*n*= 64)	45.3 (95% CI: NR)				

CNS: central nervous system; NR: not reached; LDH: Lactate dehydrogenase; I-O: Immuno-oncology; ULN: Upper limit of normal. Statistically significant values (*p* ≤ 0.05) are highlighted in bold.

## Data Availability

Data are available on reasonable request. Deidentified patient-level data can be obtained from the authors upon reasonable request.

## Supplementary Materials

The following supporting information can be downloaded at: https://www.mdpi.com/article/10.3390/cancers18121994/s1, Table S1: Association between clinical and demographic factors and objective response rate; Table S2: Univariate and multivariate Cox regression analysis according to clinical and demographic factors for PFS outcomes in the first-line treatment cohort; Table S3: Univariate and multivariate Cox regression analysis according to clinical and demographic factors for OS outcomes in the first-line treatment cohort; Table S4: Univariate and multivariate Cox regression analysis according to clinical and demographic factors for PFS outcomes in the second-line treatment cohort; Table S5: Univariate and multivariate Cox regression analysis according to clinical and demographic factors for OS outcomes in the second-line treatment cohort.

## Abbreviations

CIs	Confidential interval
CR	Complete response
I-O	Immuno-oncology
IQR	Interquartile range
irAEs	Immune-related adverse events
LDH	Lactate dehydrogenase
NR	Not reached
OS	Overall survival
PD	Progressive disease
PFS	Progression-free survival
PR	Partial response
iRECIST	Immune-Related Response Evaluation Criteria in Solid Tumors
SD	Stable disease
ULN	Upper limit of normal
