# Supplementary material for: Real-World Outcomes of Nivolumab Plus Ipilimumab in Metastatic Melanoma: A Stratified Analysis of First- and Second-Line Treatment
Source: Cancers (Basel). 2026 Jun 18;18(12):1994. doi: 10.3390/cancers18121994 (PMC13297584; doi:10.3390/cancers18121994)
Supplement: Supplementary file 1 [file cancers-18-01994-s001.zip › cancers-4249459-supplementary.pdf]

Supplementary Table S1. Association between clinical and demographic factors and objective response rate

Variables		Objective response rate		Pearson Chi-Square. p-value
		No Objective Response (n=111)	Partial Response / Complete Response (n=94)	
Age	≤55	59 (53.1%)	43 (45.7%)	0.290
	>55	52 (46.9%)	51 (54.2%)	
Sex	Male	56 (50.4%)	48 (51.0%)	0.930
	Female	55 (49.6%)	46 (48.9%)	
BRAF status	BRAFwt	47 (42.3%)	41 (43.6%)	0.482
	BRAFmut	58 (52.2%)	51 (54.2%)	
LDH	>ULN	35 (31.5%)	19 (20.2%)	0.125
	≤ULN	33 (29.7%)	38 (40.4%)	
I-O toxicity	Yes	65 (58.5%)	56 (59.6%)	0.810
	No	46 (41.4%)	37 (39.4%)	
Number of metastatic lesions	<4	42 (37.8%)	27 (28.7%)	0.169
	≥4	69 (62.1%)	67 (71.3%)	
I-O adjuvant/ metastatic therapy	Yes	30 (27.0%)	19 (20.2%)	0.254
	No	81 (73.0%)	75 (79.8%)	
Disease stage at initial diagnosis	Local	84 (75.6%)	57 (60.6%)	**0.021**
	Metastatic	27 (24.4%)	37 (39.4%)	

LDH: Lactate dehydrogenase; I-O: Immuno-oncology; ULN: Upper limit of normal. Statistically significant values ($p \leq 0.05$) are highlighted in bold.

Supplementary Table S2. Univariate and multivariate Cox regression analysis according to clinical and demographic factors for PFS outcomes in the first-line treatment cohort

Variables		Median PFS, mos (95% CI)	Univariable analysis		Multivariable analysis	
			HR (95% CI)	P-value	HR (95% CI)	P-value
Age	≥57 (n=72)	11.1 (6.32-15.8)	1	0.677		
	<57 (n=69)	7.9 (0.70-15.1)	1.09 (0.72-1.65)			
Sex	Male (n=69)	11.1 (4.8-17.4)	1	0.993		
	Female (n=72)	8.43 (3.7-13.1)	1.00 (0.66-1.52)			
BRAF status	BRAFwt (n=63)	12.56 (6.94-18.1)	1	**0.037**	1	**0.016**
	BRAFmut (n=71)	6.50 (2.71 – 10.28)	1.45 (1.02-2.05)		1.52 (1.08-2.14)	
LDH	≤ULN (n=47)	12.56 (0.36-24.77)	1	**0.042**	1	**0.041**
	>ULN (n=39)	4.70 (0-9.77)	1.29 (1.01 – 1.65)		1.30 (1.01-1.67)	
I-O toxicity	No (n=54)	6.50 (0.0 – 14.72)	1	0.103	1	**0.043**
	Yes (n=87)	11.16 (5.60 – 16.73)	0.70 (0.46-1.07)		0.64 (0.41-0.99)	
Number of metastatic lesions	<4 (n=98)	11.23 (6.81-15.65)	1	0.134	1	0.058
	≥4 (n=43)	4.53 (3.41-5.65)	1.40 (0.90-2.17)		1.56 (0.98-2.48)	
CNS metastasis at baseline	No (n=99)	11.23 (5.35 – 17.19)	1	0.438		
	Yes (n=41)	6.67 (3.85 – 9.49)	1.19 (0.76-1.87)			
I-O adjuvant/ metastatic therapy	No (n=125)	11.16 (2.36 – 6.53)	1	0.233	1	**0.034**
	Yes (n=16)	7.33 (4.72 – 9.94)	1.42 (0.80-2.52)		1.91 (1.05-3.48)	
Disease stage at initial diagnosis	Local (n=95)	7.9 (3.31 – 12.48)	1	0.404		
	Metastatic (n=46)	11.1 (5.53 – 16.66)	0.82 (0.52-1.30)			

LDH: Lactate dehydrogenase; I-O: Immuno-oncology; ULN: Upper limit of normal. Statistically significant values ($p \leq 0.05$) are highlighted in bold.

Supplementary Table S3. Univariate and multivariate Cox regression analysis according to clinical and demographic factors for OS outcomes in the first-line treatment cohort

Variables		Median OS, mos (95% CI)	Univariable analysis		Multivariable analysis	
			HR (95% CI)	P-value	HR (95% CI)	P-value
Age	<57 (n=69)	NR	1	0.111	1	0.079
	≥57 (n=72)	29.2 (2.4-55.7)	1.59 (0.89 – 2.82)		1.68 (0.94-3.03)	
Sex	Male (n=69)	NR	1	0.281	1	**0.036**
	Female (n=72)	29.2 (95% CI: NR)	1.36 (0.78-2.34)		1.91 (1.04-3.52)	
BRAF status	BRAFwt (n=63)	29.1 (95% CI: NR)	1	0.633		
	BRAFmut (n=71)	NR	0.88 (0.54-1.46)			
LDH	≤ULN (n=47)	NR	1	0.311		
	>ULN (n=39)	52.48 (1.32 – 103.60)	1.18 (0.85-1.64)			
I-O toxicity	No (n=54)	52.46 (0.0 – 110.231)	1	0.295	1	0.051
	Yes (n=87)	NR	0.74 (0.42-1.30)		0.54 (0.29-1.00)	
Number of metastatic lesions	<4 (n=98)	NR	1	**0.042**	1	**0.005**
	≥4 (n=43)	24.16 (0.0 – 53.1)	1.79 (1.02-3.14)		2.32 (1.28-4.19)	
CNS metastasis at baseline	No (n=99)	NR	1	0.816		
	Yes (n=41)	NR	0.93 (0.50-1.72)			
I-O adjuvant/ metastatic therapy	No (n=125)	NR	1	0.697		
	Yes (n=16)	28.43 (95% CI: NR)	1.17 (0.53-2.61)			
Disease stage at initial diagnosis	Local (n=95)	52.47 (17.85-87.08)	1	0.827		
	Metastatic (n=46)	NR	0.93 (0.51-1.71)			

LDH: Lactate dehydrogenase; I-O: Immuno-oncology; ULN: Upper limit of normal; NR: not reached. Statistically significant values (p ≤ 0.05) are highlighted in bold.

Supplementary Table S4. Univariate and multivariate Cox regression analysis according to clinical and demographic factors for PFS outcomes in the second-line treatment cohort

Variables		Median PFS, mos (95% CI)	Univariable analysis		Multivariable analysis	
			HR (95% CI)	P-value	HR (95% CI)	P-value
Age	<57 (n=32)	6.1 (3.4-8.8)	1	0.491	1	**0.037**
	≥57 (n=31)	5.6 (0.1-13.6)	1.23 (0.68-2.22)		2.04 (1.04-3.99)	
Sex	Мужской (n=35)	7.1 (0.1-15.6)	1	0.239		
	Женский (n=28)	2.9 (0.1-5.6)	1.42 (0.79-2.55)			
BRAF status	BRAFwt (n=25)	2.2 (1.17-9.98)	1	0.573		
	BRAFmut (n=37)	7.1 (0.1-20.1)	0.85 (0.47-1.51)			
LDH	>ULN (n=14)	2.7 (0.1-7.3)	1	0.230	1	**0.012**
	≤ULN (n=24)	3.4 (0.1-7.48)	0.82 (0.59-1.13)		0.63 (0.44-0.90)	
I-O toxicity	No (n=29)	3.8 (1.1-6.6)	1	0.504		
	Yes (n=34)	6.2 (2.8-9.7)	0.82 (0.45-1.47)			
Number of metastatic lesions	≥4 (n=25)	6.2 (1.12-11.33)	1	0.852	1	**0.045**
	<4 (n=38)	5.5 (2.65-8.48)	0.94 (0.52-1.71)		0.48 (0.24-0.98)	
CNS metastasis at baseline	No (n=35)	17.8 (0.1-49.0)	1	**<0.001**	1	<0.001
	Yes (n=28)	2.6 (1.04-4.15)	3.03 (1.65-5.56)		8.40 (3.57-19.78)	
I-O adjuvant/ metastatic therapy	No (n=37)	4.9 (0.1-32.4)	1	0.427		
	Yes (n=26)	6.1 (3.9-8.3)	1.27 (0.71-2.28)			
Disease stage at initial diagnosis	Local (n=45)	4.3 (1.67-6.92)	1	0.155	1	0.065
	Metastatic (n=18)	10.3 (0.1-23.07)	0.61 (0.31-1.20)		0.51 (0.25-1.04)	

LDH: Lactate dehydrogenase; I-O: Immuno-oncology; ULN: Upper limit of normal. Statistically significant values ($p \leq 0.05$) are highlighted in bold.

Supplementary Table S5. Univariate and multivariate Cox regression analysis according to clinical and demographic factors for OS outcomes in the second-line treatment cohort

Variables		Median OS, mos (95% CI)	Univariable analysis		Multivariable analysis	
			HR (95% CI)	P-value	HR (95% CI)	P-value
Age	≥52 (n=31)	30.5 (95% CI: NR)	1	0.743		
	<52 (n=32)	21.7 (95% CI: NR)	1.13 (0.54-2.35)			
Sex	Male (n=35)	NR	1	0.374		
	Female (n=28)	25.5 (11.8-39.0)	1.39 (0.67-2.89)			
BRAF status	BRAFwt (n=25)	25.5 (95% CI: NR)	1	0.689		
	BRAFmut (n=37)	NR	1.15 (0.57-2.34)			
LDH	≤ULN (n=24)	NR	1	0.768		
	>ULN (n=14)	7.9 (0.1-26.9)	1.06 (0.71-1.60)			
I-O toxicity	No (n=29)	13.8 (0.1-45.3)	1	0.164		
	Yes (n=34)	NR	0.59 (0.27-1.24)			
Number of metastatic lesions	<4 (n=38)	NR	1	0.979		
	≥4 (n=25)	7.6 (15.7-45.3)	1.01 (0.48-2.12)			
CNS metastasis at baseline	No (n=35)	NR	1	**0.002**	1	**0.002**
	Yes (n=28)	5.41 (0.1-20.2)	3.32 (1.54-7.17)		3.23 (1.54-7.19)	
I-O adjuvant/ metastatic therapy	No (n=37)	17.4 (0.1-46.2)	1	0.390		
	Yes (n=26)	NR	0.72 (0.34-1.51)			
Disease stage at initial diagnosis	Local (n=45)	21.7 (3.1-40.3)	1	0.140	1	0.092
	Metastatic (n=18)	NR	0.49 (0.18-1.27)		0.43 (0.16-1.14)	

LDH: Lactate dehydrogenase; I-O: Immuno-oncology; ULN: Upper limit of normal; NR: not reached. Statistically significant values ($p \leq 0.05$) are highlighted in bold.
